# Combination of rs‐fMRI, QSM, and ASL Reveals the Cerebral Neurovascular Coupling Dysfunction Is Associated With Cognitive Decline in Patients With Chronic Kidney Disease

**DOI:** 10.1111/cns.70151

**Published:** 2024-12-05

**Authors:** Lijun Song, Hao Wang, Wenbo Yang, Mingan Li, Boyan Xu, Min Li, Heyu Ding, Han Lv, Pengfei Zhao, Zhenghan Yang, Wenhu Liu, Zhen‐chang Wang, Xu Liu

**Affiliations:** ^1^ Department of Radiology, Beijing Friendship Hospital Capital Medical University Beijing China; ^2^ MR Research GE Healthcare Beijing China; ^3^ Clinical Epidemiology and EBM Unit, Beijing Friendship Hospital Capital Medical University Beijing China; ^4^ Department of Nephrology, Beijing Friendship Hospital Capital Medical University Beijing China

**Keywords:** arterial spin labeling, chronic kidney disease, cognitive decline, neurovascular coupling, quantitative susceptibility mapping

## Abstract

**Background:**

Neurovascular coupling (NVC) reflects the close connection between neural activity and cerebral blood flow (CBF) responses, providing new insights to explore the neuropathological mechanisms of various diseases. Non‐dialysis patients with chronic kidney disease (CKD) exhibit cognitive decline, but the underlying pathological mechanisms are unclear.

**Methods:**

The prospective study involved 53 patients with stage 1–3a CKD (CKD1–3a), 78 patients with stage 3b–5 CKD (CKD3b–5), and 52 healthy controls (HC). Our investigation involved voxel‐based assessments of both global and regional BOLD signal characteristics. Additionally, we explored the correlations between neuroimaging indices, Montreal Cognitive Assessment (MoCA) scores, and clinical laboratory findings.

**Results:**

Compared to HC, the CKD3b–5 and CKD1–3a groups exhibited lower ALLF and ReHo in the default mode network (DMN), higher CBF in bilateral hippocampus (HIP), higher susceptibility values in bilateral caudate nucleus (CAU) and putamen (PUT), and lower susceptibility values in bilateral HIP. At the global level, the coupling coefficients were lower in CKD1–3a and CKD3b–5 groups than in HC. At the ROI level, the CBF‐ALFF and CBF‐ReHo coupling in HIP and basal ganglia regions were lower in CKD3b–5 groups than in the CKD1–3a group. Most importantly, susceptibility‐ALFF in ANG.R may mediate the effects of phosphorus on cognitive decompensation in patients with CKD1‐3a.

**Conclusions:**

Non‐dialysis patients with CKD exhibit abnormal NCV, which is associated with the cognitive decline. Specifically, the susceptibility‐ALFF may serve as a valuable biomarker for early assessment of cognitive decline in CKD, offering insights into the pathogenesis of cognitive decline in CKD.

## Graphical abstract

Susceptibility‐ALFF may serve as a valuable biomarker for early assessment of cognitive decline in CKD, offering insights into the pathogenesis of cognitive decline in CKD. HC, healthy control; ALFF, amplitude of low‐frequency fluctuations; ReHo, regional homogeneity; CBF, cerebral blood flow; ROI, region of interest.

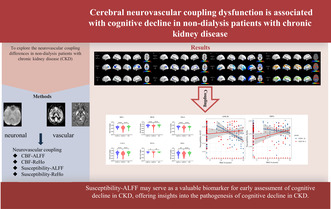

## 1 Introduction

CKD, defined as chronic renal structural and functional dysfunction (history of renal damage > 3 months) from a variety of causes, including pathologically normal or abnormal renal GFR impairment, abnormal blood or urine composition, abnormal imaging findings, or unexplained decline in GFR (< 60 mL/min·1.73 m^2^) for more than 3 months [1], has emerged as a pressing global public health concern [2, 3, 4]. Notably, individuals with CKD experience cognitive decline [5], including encephalopathy, confusion, and dementia [6], affecting 10%–40% of these patients [7], with a particularly pronounced impact on those in CKD stage 5 [8]. Hemodialysis (HD) is the primary renal replacement therapy for patients with stage 5 CKD, defined as eGFR < 15 mL/min/1.73 m^2^ [9]. Although HD has been shown to improve the working memory of patients [10], hemodynamic challenges associated with HD, including dialysis‐induced hypoperfusion, can further exacerbate cognitive decline [11]. Thus, there is an urgent imperative to explore the underlying neuropathological mechanisms of cognitive decline in non‐dialysis patients with CKD and identify potential biomarkers associated with this phenomenon.

Functional magnetic resonance imaging (fMRI), a noninvasive and dependable technique for comprehensively assessing brain function [12], has gained substantial traction in recent years as a pivotal tool for exploring the pathological mechanisms of cognitive impairment in patients with CKD [13, 14]. Recently, a series of resting‐state fMRI (rs‐fMRI) studies showed abnormalities in ALFF and ReHo values within the default mode network (DMN) region among patients with CKD. These findings revealed abnormal spontaneous neural activity in patients with CKD, establishing a vital foundation for understanding cognitive decline in this population [13, 15, 16, 17]. In addition, several studies showed significant reduction in CBF associated with cognitive scores in patients with stage 5 CKD after the dialysis initiation. It highlights the potential contribution of reduced cerebral perfusion to the observed cognitive decline [18, 19]. However, these studies predominantly focused on single imaging modalities, which may not holistically capture the complexities of neurological function changes induced by CKD. Therefore, the integration of multimodal neuroimaging approaches has emerged as a valuable tool to comprehensively explore the neuropathological mechanisms underlying various diseases.

In one of our own studies, the combination of arterial spin labeling (ASL) and quantitative susceptibility mapping (QSM) unveiled dysfunction in the coupling of CBF and susceptibility values within the bilateral hippocampus (HIP) among non‐dialysis patients with CKD, which correlated with cognitive decline [20]. It is important to recognize that neurons and blood vessels together constitute a functional complex known as the neurovascular unit (NVU) [21]. NVC elucidates the close, temporal, and regional connection between neural activity and CBF responses, serving as the mechanism by which the NVU regulates CBF. This coupling ensures the adequate supply of blood to neurons to meet their energy demands during activity [22]. The utilization of rs‐fMRI and ASL imaging to investigate NVC plays a pivotal role in exploring the neuropathological mechanisms of various diseases [23, 24, 25, 26, 27]. However, to the best of our knowledge, there have been no studies on NVC in non‐dialysis patients with CKD.

In addition, another of our studies revealed that increased iron deposition in the putamen (PUT) was accompanied by decreased CBF in patients on maintenance HD [14]. Vascular injuries and dysfunction may be the pivotal contributors to altered cerebral perfusion and perturbed cerebral iron metabolism [14]. The assessment of vascular status through the combination of ASL and QSM may offer fresh perspectives on NVC. Therefore, this study aimed to explore NVC differences in non‐dialysis patients with CKD, and we formulated the following hypotheses: (1) There exist regional and global variations in spontaneous neural activity (ALFF and ReHo), iron deposition, and CBF in patients with CKD. (2) NVC is significantly altered at both global and localized levels in patients with CKD. (3) Changes in spontaneous neural activity, iron deposition, CBF, and NVC are correlated with biochemical parameters and Montreal Cognitive Assessment (MoCA) scores.

## 2 Materials and Methods

### 2.1 Subjects and Clinical Data

**FIGURE 1:**
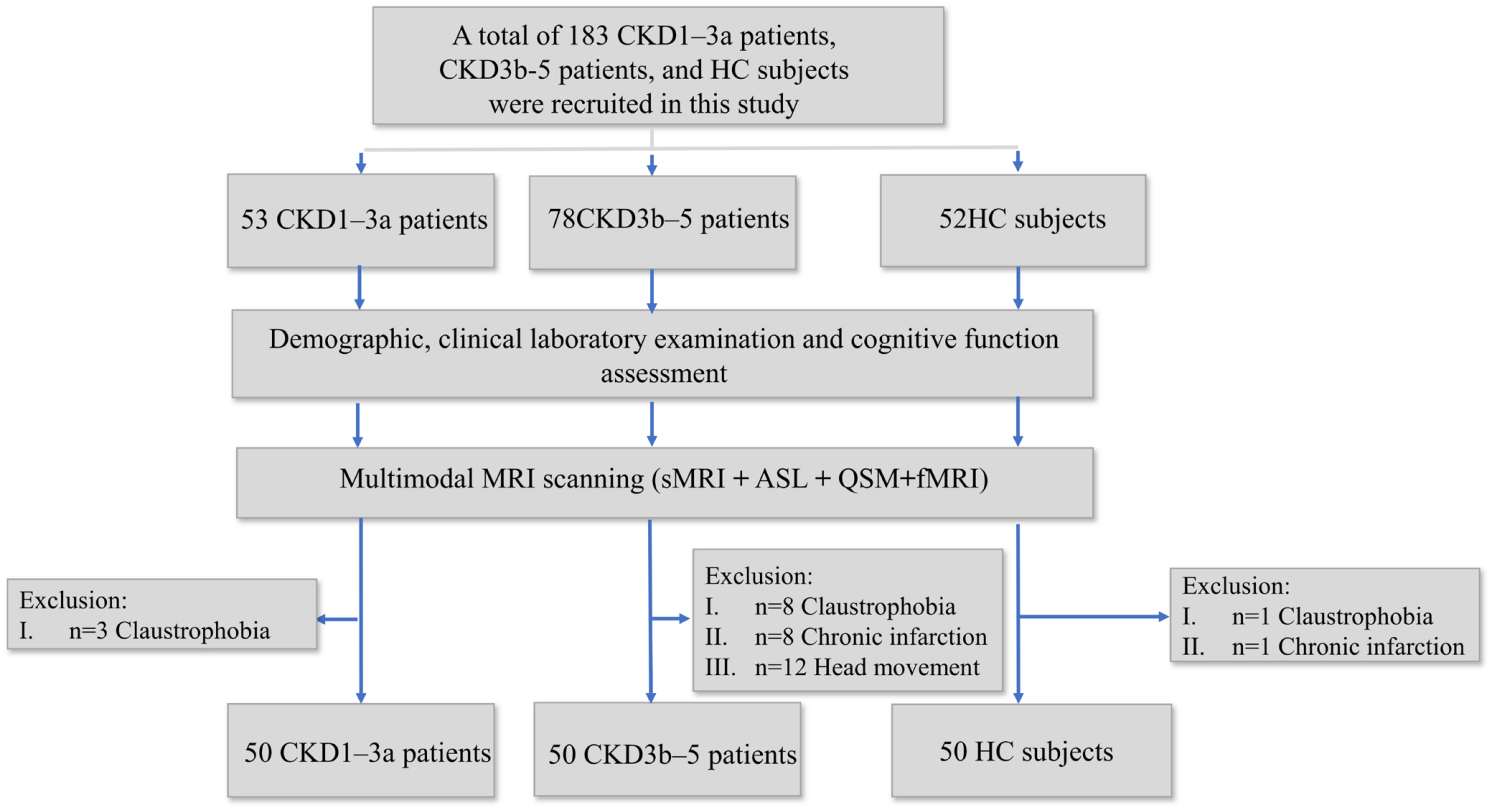
Summary of patient recruitment and exclusions. ASL, arterial spin labeling; CKD, chronic kidney disease; CKD1–3a, patients with stage 1–3a chronic kidney disease; CKD3b–5, patients with stage 3b–5 chronic kidney disease; fMRI, functional magnetic resonance imaging; HC, healthy control; QSM, quantitative susceptibility mapping; sMRI, structural MRI.

From June 2023 to January 2024, a total of 53 patients with CKD1–3a and 78 patients with CKD3b–5 from the Department of Nephrology, Beijing Friendship Hospital, Capital Medical University were recruited for this study. In addition, 52 sex‐, age‐, and education‐matched healthy volunteers from local community districts were recruited. The study was approved by the Ethics Committee of Beijing Friendship Hospital, Capital Medical University, and was conducted in accordance with the Declaration of Helsinki. Each participant provided written informed consent. Inclusion criteria included (1) 18–65 years of age, (2) right‐handed, (3) patients diagnosed with CKD according to the National Kidney Foundation—Kidney Disease Outcomes Quality Initiative guidelines [28, 29], and (3) patients with glomerulonephritis. Exclusion criteria were as follows: (1) contraindications for magnetic resonance imaging; (2) history of dialysis treatment; (3) history of neurologic disorders, including stroke, cerebral infarct, brain tumors, and traumatic brain injury; (4) history of psychiatric disorders; (5) history of diabetes mellitus; and (6) significant head motion during MRI scanning (≥ 3 mm/3°). Ultimately, 12, 9, and 12 participants were excluded due to claustrophobia, chronic infarction, and head movement, respectively. Details of the study participants are shown in Figure 1. Prior to scanning, all patients with CKD1–3a and patients with CKD3b–5 underwent MoCA and laboratory biochemistry.

### 2.2 Multimodal MRI Data Acquisition

For each participant, rs‐fMRI data, high‐resolution T1‐weighted imaging, ASL, and QSM data were obtained sequentially using a 3.0 T magnetic resonance scanner (Discovery MR750w, General Electric, USA) with an eight‐channel phased array coil. Detailed scanning parameters can be found in the Data S1.

### 2.3 Multimodal Data Analysis

**FIGURE 2:**
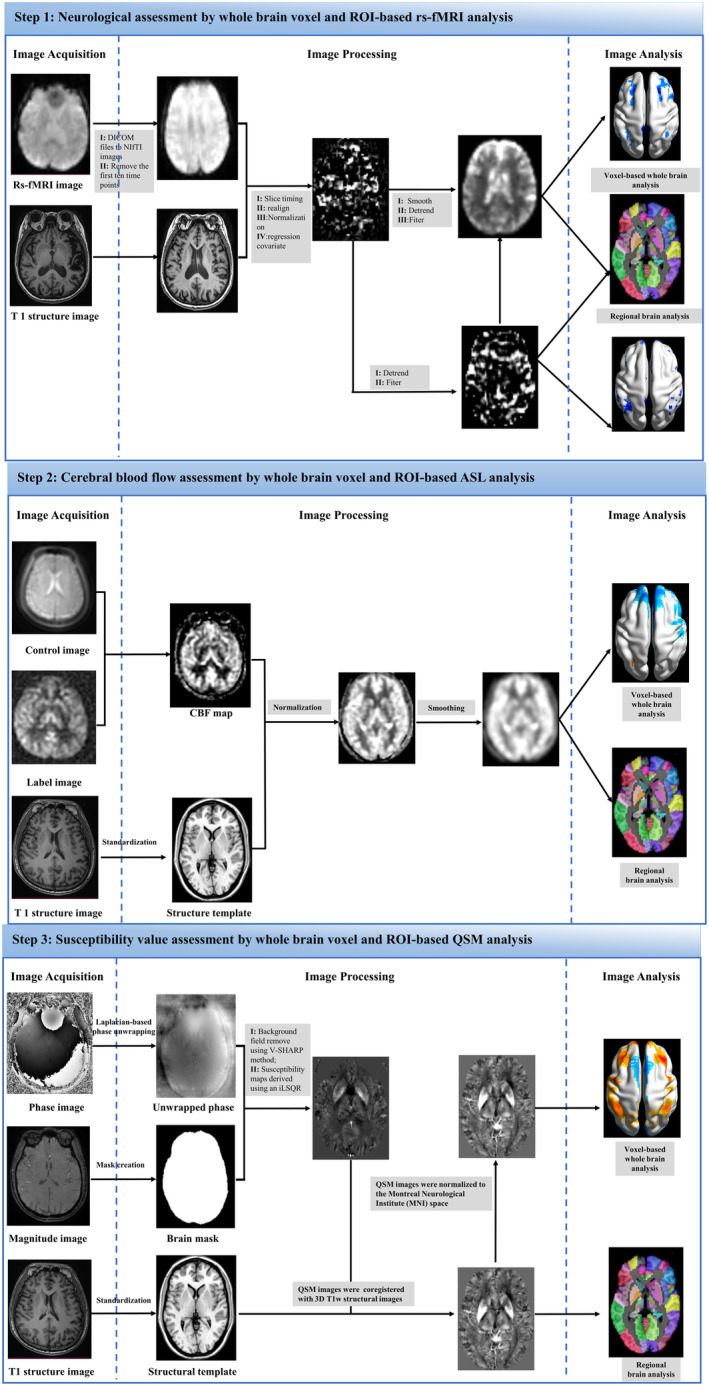
Flowchart of the experiment. ALFF, amplitude of low‐frequency fluctuation; CBF, cerebral blood flow; QSM, quantitative susceptibility mapping; ReHo, regional homogeneity; ROI, region of interest; rs‐fMRI, resting‐state functional magnetic resonance imaging.

Data processing was accomplished using DPABI V6.0 (http://rfmri.org/dpabi), SPM12, STI Suite version 3.0 package, and code written in MATLAB 2018b (MathWorks, Natick, MA). The data processing flow is shown in Figure 2. Detailed data preprocessing can be found in the Data S1.

### 2.4 Mediation Analysis

To investigate the effect of clinical blood biochemical indices on MoCA and the altered patterns of NVC in patients with CKD, we performed a mediational analysis; details are provided in the Data S1.

### 2.5 Statistical Analyses

All statistical analyses were performed using SPSS 25.0 software and GraphPad Prism Version 9.3.0. Normality of ALFF, ReHo, CBF, and susceptibility values were assessed using the Kolmogorov–Smirnov test. The Kruskal–Wallis test was used to test for between‐group differences in age and education, and the Chi‐square test was used to test between‐group differences in sex and medication. Further, the Mann–Whitney *U*‐test was used to test laboratory test results (urea, creatinine, phosphate, blood Ca, uric acid, and blood albumin) between CKD1–3a group and CKD3b–5 groups. A two‐sample t test was used to test for between‐group differences in hemoglobin. Multiple linear regression analysis was used to eliminate the effects of sex, age, education, medication, and total intracranial volume (TIV) on the MoCA differences between groups. A voxel‐wise t test with sex, age, education, medication, and TIV as covariates was used to test for between‐group differences in ALFF, ReHo, CBF, and susceptibility values, and an FDR correction with a significance threshold of *p* < 0.05 was used.

Global and ROI NVC coefficients were examined using the Kruskal–Wallis test. Mixed linear modeling was used to eliminate the effects of sex, age, education, medication, and TIV on differences in NVC among groups. A partial correlation analysis with sex, age, education level, medication, and TIV as covariates was used to determine the relationship between ALFF, ReHo, CBF, susceptibility values, and NVC values and MoCA scores in the three groups. For multiple comparisons, the Bonferroni method (*p* < 0.05/116 = 0.0004) was used to correct the tests.

## 3 Results

### 3.1 Demographic and Clinical Characteristics

The demographic and clinical characteristics of the participants are shown in Table 1. No significant differences were observed among CKD1–3a, CKD3b–5, and HC in terms of age (*p* = 0.990), sex (*p* = 0.509), and educational level (*p* = 0.266). In addition, there were significant differences in urea, creatinine, phosphorus, calcium, hemoglobin, blood albumin, eGFR, hypertension history, and medication between the CKD1–3a and CKD3b–5 groups. MoCA scores were significantly lower in the CKD3b–5 group than in the CKD1–3a group (*p* = 0.017; Table 2).

**TABLE 1:** Demographic and clinical characteristics of the participants.

	CKD1–3a (*n* = 50)	CKD3b–5 (*n* = 50)	HC (*n* = 50)	*p*
Age (years)^e^	54.0 (39.8, 61)	53.5 (43.0, 60.0)	54.0 (44.8, 59.3)	0.990^a^
Sex (male/female)	32/18	32/18	29/21	0.509^b^
Education (years)^e^	12.0 (11.3, 16.0)	12.0 (9.0, 15.0)	12.0 (12.0, 16.0)	0.266^a^
Urea (mmol/L)^e^	5.9 (4.3, 7.2)	27.4 (17.5, 34.5)	NA	**< 0.0001** ^d^
Creatinine (μmol/L)^e^	89.1 (68.4, 114.0)	643.6 (507.0, 933.9)	NA	**< 0.0001** ^d^
Phosphorus (mmol/L)^e^	1.2 (1.1, 1.4)	2.0 (1.5, 2.4)	NA	**< 0.0001** ^d^
Ca (mmol/L)^e^	2.2 (2.1, 2.3)	2.1 (2.0, 2.3)	NA	0.430^d^
Hemoglobin (g/L)^f^	130.3 ± 20.8	100.2 ± 19.4	NA	**< 0.0001** ^c^
Uric acid (μmol/L)^e^	399.7 (339.2, 477.6)	412.7 (351.1, 533.8)	NA	0.558^d^
Blood albumin (g/L)^e^	33.1 (27.8, 38.2)	36.9 (33.9, 39.3)	NA	**0.011** ^d^
eGFR (mL/min)^e^	85.4 (62.9, 102.6)	6.9 (5.0, 10.3)	NA	**< 0.0001** ^d^
Hypertension history (yes/no)	33/17	43/7	NA	**0.019** ^**b**^
Medication				
EPO (yes/no)	6/44	35/15	NA	**< 0.001** ^b^
Vitamin D3 (yes/no)	7/43	21/29	NA	**0.002** ^b^
ACEI (yes/no)	33/17	8/42	NA	**< 0.001** ^b^

*Note:* Bold values indicates statistically significant difference of *p* values.

Abbreviations: ACEI, angiotensin‐converting enzyme inhibitor; CKD1–3a, patients with stage 1–3a chronic kidney disease; CKD3b–5, patients with stage 3b–5 chronic kidney disease; EPO, erythropoietin; HC, healthy control; NA, not applicable.

^a^ Kruskal–Wallis test.

^b^ Chi‐square test.

^c^ Two‐sample *t* tests.

^d^ Mann–Whitney *U*‐test.

^e^ Data are presented as median (25th–75th percentile).

^f^ Data are presented as mean ± SD.

**TABLE 2:** Neuropsychological assessment in CKD patients.

	CKD1–3a (*n* = 50)	CKD3b–5 (*n* = 50)	*β*	*p*
MoCA scores	26.0 (24.0,28.0)	24.0 (21.8, 27.0)	−1.400	**< 0.017** ^a^
Visuospatial	4.0 (4.0, 5.0)	24.0 (22.0, 27.0)	−0.48	**0.006** ^a^
Naming	3.0 (30, 3.0)	3.0 (3.0, 3.0)	−0.51	0.445^a^
Attention	6.0 (5.0,6.0)	6.0 (5.0, 6.0)	−0.264	0.103^a^
Language	3.0 (2.0, 3.0)	3.0 (2.0, 3.0)	−0.59	0.604^a^
Abstraction	2.0 (1.0, 2.0)	2.0 (1.0, 2.0)	0.064	0.525^a^
Delayed recall	3.0 (2.0, 4.0)	3.0 (1.8, 4.0)	−0.16	0.522^a^
Orientation	6.0 (6.0, 6.0)	6.0 (6.0, 6.0)	−0.155	0.165^a^

*Note:* Multiple linear regression was used with age, sex, education‐level medication, and TIV as covariates. Bold values indicates statistically significant difference of *p* values.

Abbreviations: CKD1–3a, patients with stage 1–3a chronic kidney disease; CKD3b–5, patients with stage 3b–5 chronic kidney disease; MoCA scores, Montreal Cognitive Assessment scores; TIV, total intracranial volume.

^a^ Mann–Whitney *U*‐test.

### 3.2 Global ALFF and ReHo Changes in Patients With CKD1–3a or CKD3b–5

**FIGURE 3:**
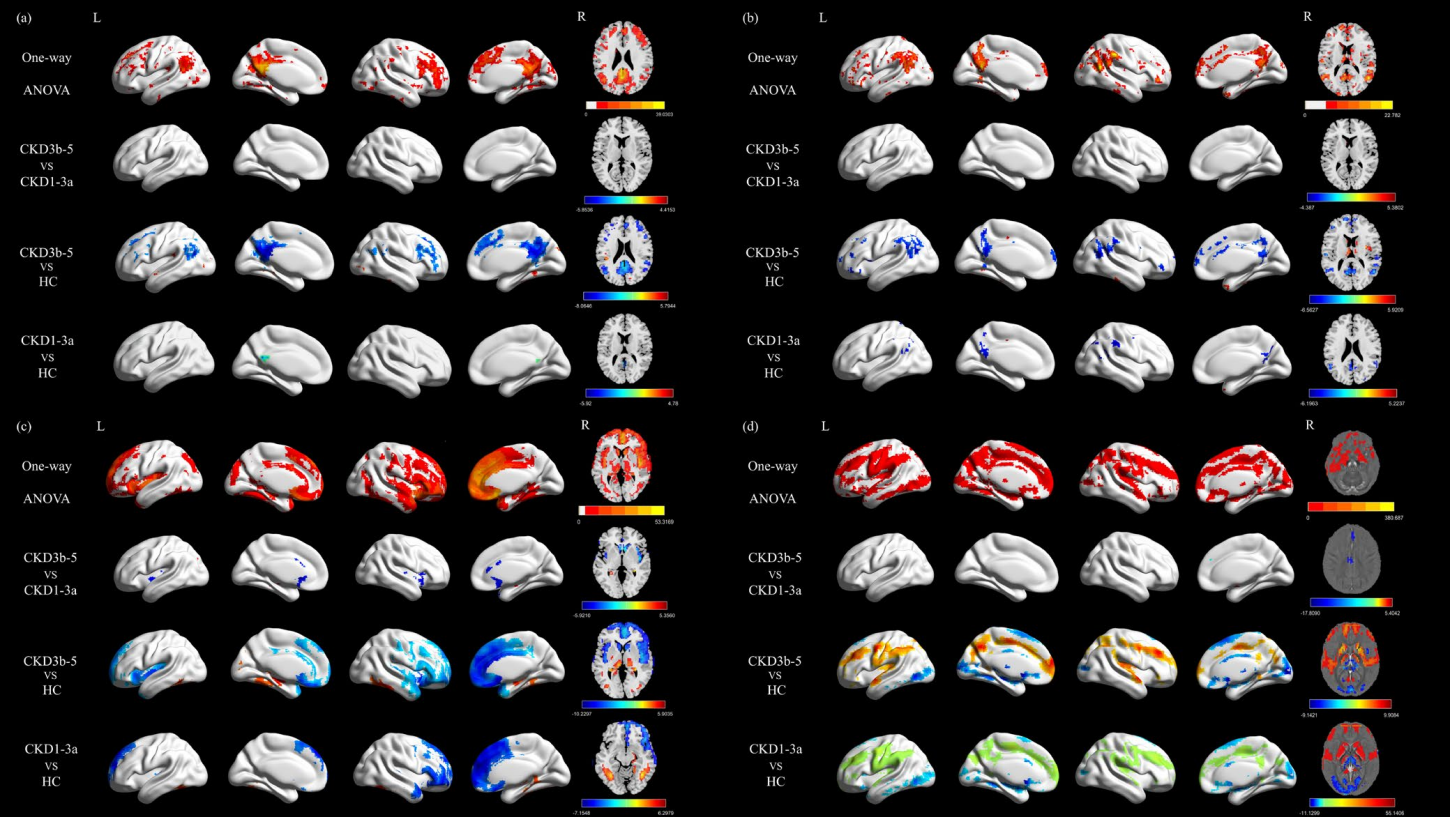
Voxel‐based analysis among CKD1–3a patients, CKD3b–5 patients, and HC. The results were shown on the study‐wise magnitude template in the MNI coordinate system. (a) Voxel‐based ALFF analysis among CKD1–3a patients, CKD3b–5 patients, and HC (*p* < 0.05, FDR corrected). (b) Voxel‐based ReHo analysis among CKD1–3a patients, CKD3b–5 patients, and HC (*p* < 0.05, FDR corrected). (c) Voxel‐based CBF analysis among CKD1–3a patients, CKD3b–5 patients, and HC (*p* < 0.05, FDR‐corrected). (d) Voxel‐based QSM analysis among CKD1–3a patients, CKD3b–5 patients, and HC (*p* < 0.05, FDR corrected). ALFF, amplitude of low‐frequency fluctuations; CBF, cerebral blood flow; CKD, chronic kidney disease; CKD1–3a, patients with stage 1–3a chronic kidney disease; CKD3b–5, patients with stage 3b–5 chronic kidney disease; FDR, false discovery rate; HC, healthy control; MNI, Montreal Neurological Institute; QSM, quantitative susceptibility mapping; ReHo, regional homogeneity.

ALFF, ReHO, CBF, and susceptibility spatial distribution maps were consistent among the three groups (Figure S1). ANOVA results showed statistically significant differences in ALFF, ReHO, CBF, and susceptibility value among the three groups (Figure 3). Compared with HC, patients with CKD3b–5 showed higher ALFF in right fusiform (FFG), bilateral middle occipital gyrus (MOG), right inferior temporal gyrus (ITG) and left superior temporal gyrus (STG), and lower ALLF in right precuneus (PCUN), bilateral middle frontal gyrus (MFG), bilateral inferior parietal gyrus (IPL), and right superior temporal gyrus (STG) and left inferior frontal gyrus, triangular part (IFGtriang). Compared with HC, patients with CKD1–3a showed lower ALFF in left PCUN (*p* < 0.05, cluster‐level FDR‐corrected; Figure 3a and Table 3). As for ReHo, compared with HC, patients with CKD3b–5 showed higher ReHo in right thalamus (THA), right insula (INS), right caudate nucleus (CAU), and right ITG, and lower ReHo in left PUT, right middle temporal gyrus (MTG), left superior frontal gyrus, dorsolateral (SFGdor), left IPL, and left PCUN. Compared with HC, patients with CKD1–3a showed lower ReHo in left PCUN, right STG, and left MOG (*p* < 0.05, cluster‐level FDR‐corrected; Figure 3b and Table 3).

**TABLE 3:** Brain regions with differences in ALFF or ReHo values between groups.

Brain regions	Cluster size	MNI coordinate			Peak *t* value
		*x*	*y*	*z*	
ALFF					
CKD 3b–5 > HC					
Right fusiform	35	27	−48	−12	5.33
Left middle occipital gyrus	45	−33	−96	9	4.28
Right middle occipital gyrus	50	36	−93	9	4.98
Right inferior temporal gyrus	39	51	−36	−27	5.21
Left superior temporal gyrus	21	−45	−33	24	5.79
CKD 3b–5 < HC					
Right middle frontal gyrus	861	24	24	39	−7.96
Left middle frontal gyrus	415	−21	27	36	−7.13
Right precuneus	1486	−3	−39	24	−8.06
Right superior temporal gyrus	108	51	−42	21	−5.26
Left inferior parietal gyrus	49	−30	−48	39	−5.55
Right inferior parietal gyrus	39	39	−54	42	−4.61
Left inferior frontal gyrus, triangular part	26	−48	30	12	−4.50
CKD 1–3a < HC					
Left precuneus	24	−3	−51	15	−5.68
ReHo					
CKD 3b–5 > HC					
Right thalamus	18	−6	−15	15	4.64
Right insula	26	33	−3	15	5.42
Right caudate nucleus	27	15	−15	21	4.78
Right inferior temporal gyrus	49	48	−30	−27	5.92
CKD 3b–5 < HC					
Left putamen	50	−21	9	−3	−5.76
Right middle temporal gyrus	285	51	−57	18	−6.56
Left superior frontal gyrus, dorsolateral	323	−15	57	15	−5.63
Left inferior parietal gyrus	366	−39	−57	45	−6.02
Left precuneus	463	−9	−63	18	−6.10
CKD 1–3a < HC					
Left precuneus	138	−3	−57	33	−5.99
Right superior temporal gyrus	47	51	−45	21	−5.50
Left middle occipital gyrus	30	−51	−51	21	−5.17

*Note:* Brain regions with differences in ALFF or ReHo values between groups.

Abbreviations: ALFF, amplitude of low‐frequency fluctuations; CKD 3b–5, patients with stage 3b–5 chronic kidney disease; CKD1–3a, patients with stage 1–3a chronic kidney disease; HC, healthy control; MNI, Montreal Neurological Institute; ReHo, regional homogeneity.

### 3.3 Global CBF Changes in Patients With CKD1–3a or CKD3b–5

Compared with the CKD1–3a group, patients with CKD3b–5 showed higher CBF in bilateral HIP and lower CBF in right ITG, INS, and CAU. Compared with HC, patients with CKD3b–5 showed higher CBF in bilateral HIP, left PCUN, right MOG and left superior occipital gyrus (SOG), and lower CBF in right superior frontal gyrus, medial (SFGmed), left MTG, SFGdor, and IFGtriang. Compared with HC, patients with CKD1–3a showed higher CBF in right HIP and PCUN, and bilateral FFG, and lower CBF in right MFG, left INS, and right SFGmed (*p* < 0.05, cluster‐level FDR‐corrected; Figure 3c and Table 4).

**TABLE 4:** Brain regions exhibiting CBF or susceptibility value differences among groups.

Brain regions	Cluster size	MNI coordinate			Peak *t* value
		*x*	*y*	*z*	
CBF					
CKD 3b–5 > CKD 1–3a					
Left hippocampus	62	−15	−33	9	5.00
Right hippocampus	60	33	−36	0	5.36
CKD 3b–5 < CKD 1–3a					
Right inferior temporal gyrus					
Right caudate nucleus	359	6	15	0	−5.92
Right insula	203	42	−6	−3	−5.71
CKD 3b–5 > HC					
Left hippocampus	876	−12	−18	12	5.90
Right hippocampus	789	36	−33	−3	5.86
Left superior occipital gyrus	111	−15	−81	18	4.97
Left precuneus	101	−12	−60	42	4.69
Right middle occipital gyrus	55	39	−75	−6	4.64
CKD 3b–5 < HC					
Right superior frontal gyrus, medial	526	3	29	21	−10.2
Left middle temporal gyrus	81	−69	−18	−9	−4.37
Right superior frontal gyrus, dorsolateral	33	12	9	75	−3.93
Left inferior frontal gyrus, triangular part	32	−48	45	−6	−4.09
CKD 1–3a > HC					
Right hippocampus	57	21	−21	−12	4.22
Right precuneus	86	18	−45	−12	4.59
Right fusiform gyrus	356	36	−57	−9	6.30
Left fusiform gyrus	380	−36	−57	−15	5.65
CKD 1–3a < HC					
Left insula	29	−42	−21	9	−4.18
Right middle frontal gyrus	23	42	0	48	−3.72
Right superior frontal gyrus, medial	465	6	51	18	−7.15
Susceptibility value					
CKD 3b–5 < CKD 1–3a					
Right superior frontal gyrus, dorsolateral	191	30	69	6	−17.81
Left inferior temporal gyrus	79	−69	−48	−18	−11.77
CKD 3b–5 > HC					
Left inferior frontal gyrus, orbital part	77	−30	33	−6	5.21
Left precuneus	311	−12	−60	39	9.91
Right precuneus	204	15	−63	39	5.05
Left putamen	172	−24	1	12	5.17
Right putamen	191	24	3	12	7.43
Left caudate nucleus	112	−11	17	12	4.25
Right caudate nucleus	108	20	19	12	7.73
Left middle frontal gyrus	670	−44	24	39	4.40
Right middle frontal gyrus	620	39	24	36	5.62
Left superior temporal gyrus	368	−56	−9	5	4.98
Right superior temporal gyrus	313	58	−12	5	5.94
CKD 3b–5 < HC					
Left hippocampus	143	−29	−21	−12	−5.31
Right hippocampus	94	30	−42	−6	−5.61
Left thalamus	82	−4	−21	7	−7.46
Right thalamus	108	5	−21	7	−7.89
Left inferior temporal gyrus	214	−51	−38	−15	−4.69
Left middle occipital gyrus	209	−36	−84	3	−4.74
Right middle occipital gyrus	60	33	−86	9	−5.62
CKD 1–3a > HC					
Right middle frontal gyrus	673	24	69	21	55.14
Left middle frontal gyrus	639	−21	53	21	4.72
Right angular gyrus	203	60	−55	33	3.54
Left angular gyrus	80	−41	−55	44	5.50
Right putamen	168	25	6	7	3.56
Left putamen	167	−20	15	7	6.78
Right precuneus	155	22	−57	29	4.11
Left caudate nucleus	102	−16	13	12	6.98
Right caudate nucleus	132	15	15	12	6.02
Left thalamus	83	−12	−15	12	5.05
Right thalamus	59	14	−11	12	4.37
Right superior occipital gyrus	101	16	59	17	4.46
CKD 1–3a < HC					
Left hippocampus	134	22	−12	−11	−3.37
Right hippocampus	119	−24	−19	−11	−5.75
Left thalamus	40	−7	−21	−1	−5.03
Right thalamus	58	11	−21	−1	−4.45
Left middle occipital gyrus	258	−22	19	62	−3.61
Left middle temporal gyrus	225	−54	−32	−15	−5.50

*Note:* Brain regions exhibiting CBF or susceptibility value differences among groups.

Abbreviations: CBF, cerebral blood flow; CKD1–3a, patients with stage 1–3a chronic kidney disease; CKD3b–5, patients with stage 3b–5 chronic kidney disease; HC, healthy control; MNI, Montreal Neurological Institute.

### 3.4 Global Susceptibility Value Changes in Patients With CKD1–3a and CKD3b–5

Compared with CKD1–3a group, patients with CKD3b–5 showed lower susceptibility value in right SFGdor and left ITG. Compared with HC, patients with CKD3b–5 showed higher susceptibility value in bilateral PCUN, bilateral PUT, bilateral CAU, bilateral MFG, bilateral STG and left inferior frontal gyrus, orbital part (ORBinf), and lower susceptibility value in bilateral HIP, bilateral THA, bilateral MOG and left ITG. Compared with HC, patients with CKD1–3a showed higher susceptibility value in bilateral PUT, bilateral CAU, bilateral THA, bilateral angular gyrus (ANG), bilateral MFG and right SOG, and lower susceptibility value in bilateral HIP, bilateral THA and bilateral MOG (*p* < 0.05, cluster‐level FDR‐corrected; Figure 3d and Table 4).

### 3.5 Analysis of Global or ROI‐Based NVC

**FIGURE 4:**
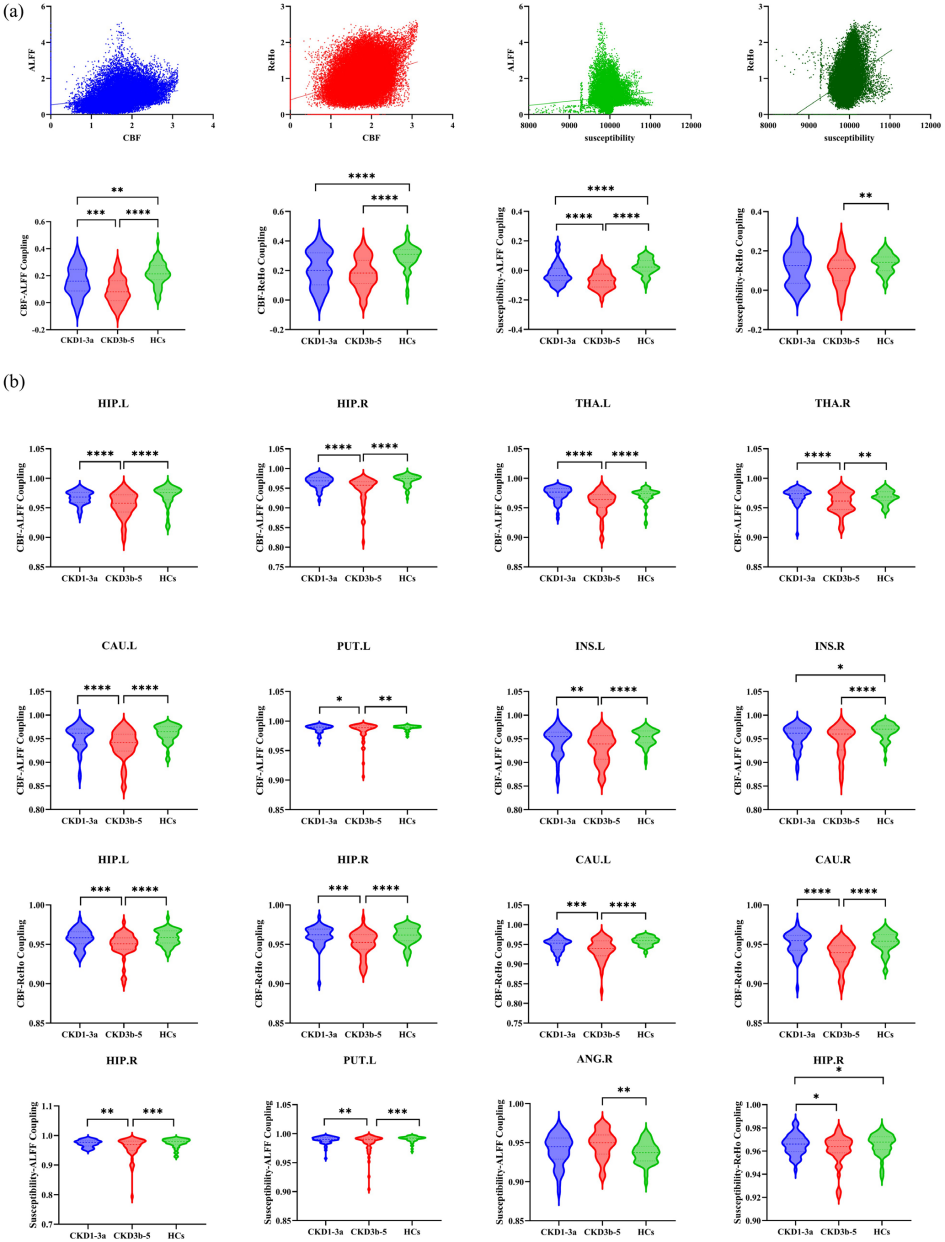
Whole‐brain and ROI‐based analysis of neurovascular coupling. (a) Whole‐brain‐based analysis of neurovascular coupling. (b) ROI‐based analysis of neurovascular coupling. *p < 0.05; **p < 0.01; ***p < 0.001; ****p < 0.0001; ALFF, amplitude of low‐frequency fluctuations; CBF, cerebral blood flow; CKD1–3a, patients with stage 1–3a chronic kidney disease; CKD3b–5, patients with stage 3b–5 chronic kidney disease; HC, healthy control; ReHo, regional homogeneity; ROI, region of interest.

At the global level, compared with the HC, both the CKD1–3a group and the CKD3b–5 group exhibited lower CBF‐ALFF coupling coefficients (*p* = 0.005, *p* < 0.0001), CBF‐ReHo coupling coefficients (*p* < 0.0001, *p* < 0.0001), and susceptibility‐ALFF coupling coefficients (*p* < 0.0001, *p* < 0.0001). The CBF‐ALFF coupling coefficients (*p* = 0.001) and susceptibility‐ALFF coupling coefficients (*p* < 0.0001) of CKD3b–5 group were lower than those of the CKD1–3a group (Figure 4a).

At the ROI level, compared with HC, patients with CKD1–3a showed lower CBF‐ALFF coupling coefficients in right INS (*p* = 0.025), patients with CKD3b–5 showed lower CBF‐ALFF coupling coefficients in bilateral HIP (*p* < 0.0001, *p* < 0.0001), bilateral THA (*p* < 0.0001, *p* = 0.004), left CAU (*p* < 0.0001), left PUT (*p* = 0.003), and bilateral INS (*p* < 0.0001, *p* < 0.0001). However, compared with CKD3b–5 group, patients with CKD1–3a showed lower CBF‐ALFF coupling coefficients in bilateral HIP (*p* < 0.0001, *p* < 0.0001), bilateral THA (*p* < 0.0001, *p* < 0.0001), left CAU (*p* < 0.0001), left PUT (*p* = 0.027), and left INS (*p* = 0.009; Figure 4b). Compared with HC, CKD3b–5 group showed lower CBF‐ReHo coupling coefficients in bilateral HIP (*p* < 0.0001, *p* < 0.0001) and bilateral CAU (*p* < 0.0001, *p* < 0.0001). However, patients with CKD1–3a showed lower CBF‐ReHo coupling coefficients in bilateral HIP (*p* = 0.001, *p* = 0.001) and bilateral CAU (*p* = 0.001, *p* < 0.0001) compared with the CKD3b–5 group. Compared with HC, the CKD3b–5 group showed lower susceptibility‐ALFF coupling coefficients in right HIP (*p* = 0.001) and left PUT (*p* = 0.001), and higher susceptibility‐ALFF coupling coefficients in right ANG (*p* = 0.006). However, patients with CKD1–3a showed lower susceptibility‐ALFF coupling coefficients in right HIP (*p* = 0.003) and left PUT (*p* = 0.01) compared with the CKD3b–5 group. Bonferroni correction was used in multiple comparisons (*p* < 0.05/116 = 0.0004; Figure 4b).

### 3.6 Correlations Between Neurovascular Indices and NVC Coefficients and Neuropsychological Assessment

At the regional level, there was a significant positive correlation between ALFF values and MoCA score in left PCUN in CKD1–3a (*r* = 0.378, *p* = 0.006) and a positive correlation between ReHo values and MoCA in left PCUN (*r* = 0.390, *p* = 0.007), bilateral ANG (*r* = 0.405, *p* = 0.005; *r* = 0.405, *p* = 0.005) in the CKD1–3a group (Figure S2a).

At the regional level, the CBF‐ALFF coupling of right anterior cingulate and paracingulate gyri (ACG) in both CKD1–3a and CKD3b–5 groups was positively correlated with MoCA (*r* = 0.311, *p* = 0.033; *r* = 0.299, *p* = 0.041). However, the susceptibility‐ReHo coupling (*r* = 0.345, *p* = 0.018; *r* = 0.045, *p* = 0.002) and CBF‐ReHo coupling (*r* = 0.511, *p* < 0.0001; *r* = 0.418, *p* = 0.003) of bilateral HIP were positively correlated with MoCA in the CKD3b–5 group (Figure S2b).

### 3.7 Mediation Analysis

**FIGURE 5:**
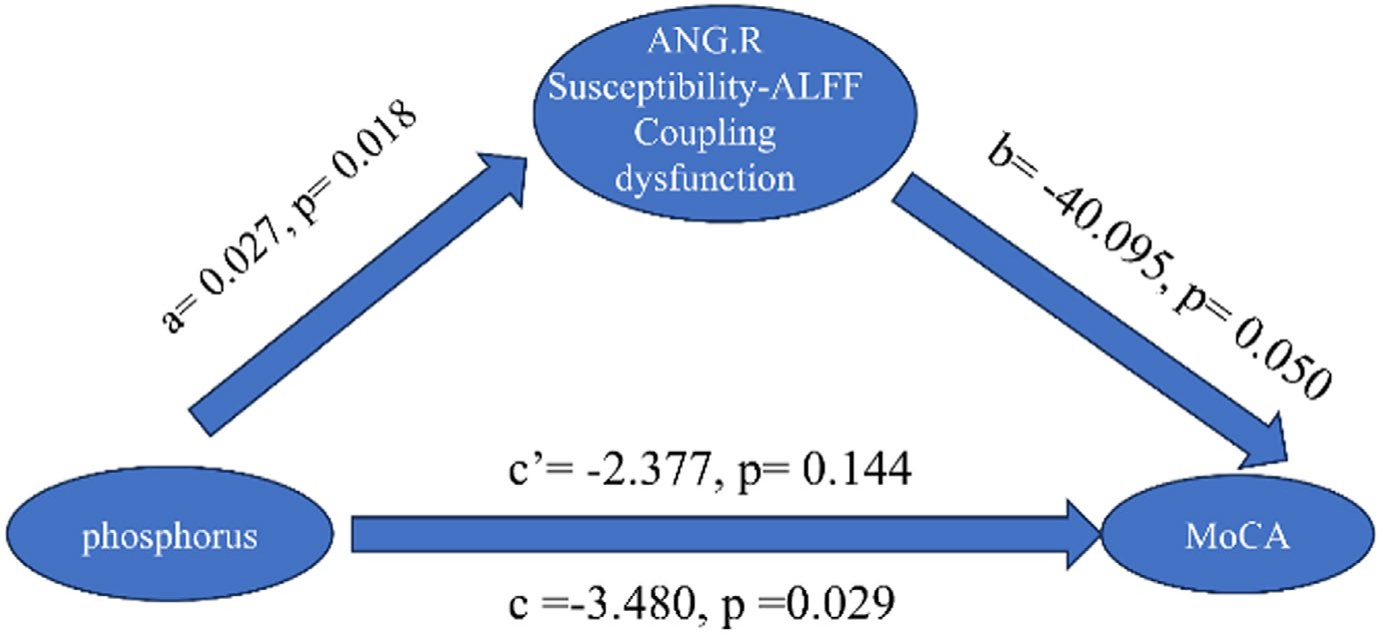
Mediation analysis. The mediation proportion effect of phosphorus on the MoCA through susceptibility‐ALFF Coupling of the ANG.R was found to be 100%. All requirements for mediating effects are met: Path *a* (*p* = 0.018) is significant, *b* (*p* = 0.050) is not significant, and the 95% Boot CI of a*b does not include the number 0 (−2.4732, −0.0585), and *c*′ is not significant. Paths a and b together represent indirect (mediating) effects. Path *c*′ is the direct effect. ANG.R, right angular gyrus; MoCA scores, Montreal Cognitive Assessment scores.

In order to investigate the relationship among the NVC pattern dysfunction, clinical blood biochemical indicators, and cognitive decline in patients with CKD, we performed mediation analyses to determine whether NVC pattern dysfunction could mediate the role of clinical indicators in cognitive decline. The results showed that higher susceptibility‐ALFF coefficients in ANG.R fully mediated the effect of phosphorus on cognitive decline in patients with CKD1–3a (c′ = −2.377, *p* = 0.14; Figure 5).

## 4 Discussion

In this study, we explored the changes in NVC and its correlations with cognitive decline in non‐dialysis patients with CKD by employing cross‐modal coupling of rs‐fMRI, ASL, and QSM. The results showed that non‐dialysis patients with CKD exhibited significant changes in ALFF, ReHo, CBF, and susceptibility values compared to HC. Importantly, both CKD 1–3a and CKD3b–5 groups exhibited reduced NVC coefficients at the global and regional levels. These findings provide the possibility to explore the relationship between the neuronal electrical activity and the corresponding vascular state based on multimodal neuroimaging.

DMN comprises a set of brain regions distributed across the parietal, temporal, and frontal cortex, which play a critical role in memory and abstract thought [30]. Previous studies showed that patients with HD exhibited significantly lower ALFF and ReHo values within the DMN compared to HC [16, 31, 32], suggesting that brain spontaneous activity is impaired in HD patients and is associated with cognitive decline. However, these studies have primarily relied on a single imaging modality without providing a comprehensive understanding of the underlying mechanisms behind cognitive decline. On the other hand, our results demonstrated that both CKD1–3a and CKD3b–5 groups displayed lower ALFF and ReHo predominantly within the DMN and a significant positive correlation between both ALFF and ReHo values of the left PCUN and MoCA scores in the CKD1–3a group. Our study suggests that even in patients with CKD1–3a, there is an early adverse impact on brain neural activity within the DMN region, indicating that CKD itself may have a detrimental effect on brain. The HIP, being part of the limbic system, is a region of great cognitive significance, playing vital roles in various cognitive pathways. In our study, we observed elevated CBF in HIP in CKD patients and significantly higher CBF values in the left HIP in the CKD3b–5 group, which is consistent with our previous findings [20]. Additionally, many central basal ganglia regions are involved in cognitive functions like procedural learning and working memory [33]. Our results showed that susceptibility values were higher in bilateral CAU and bilateral PUT and lower in bilateral HIP. This is consistent with the change in susceptibility in the basal ganglia region reported by Chai et al. [34, 35] Our findings suggest that cerebral iron deposition is altered in early CKD patients who did not receive exogenous iron absorption to treat anemia, and it may be involved in the mechanism of cognitive decline in patients with CKD.

Previous studies of the relationship between cognitive decline and NVC in patients with HD have found that HD patients exhibited reduced cerebral perfusion, altered neural activity, and disrupted NVC patterns in multiple regions [27, 36]. These studies, based on multimodal magnetic resonance methods combined with rs‐fMRI and ASL techniques, reflect the coordination between blood supply and oxygen demand during neuronal activity through NVC patterns (fALFF‐CBF, ALFF‐CBF, ReHo‐CBF, and DC‐CBF coefficients), suggesting that NVC may be a plausible mechanism contributing to cognitive decline in patients with HD [25, 27]. In addition, QSM can be used to show vascular wall structure and quantify microvascular iron deposition through blood–brain barrier damage [37]. Renowned as a noninvasive and highly precise imaging technique for quantifying brain iron content, QSM has been used in a range of neurodegenerative diseases, including Alzheimer's disease [38], Parkinson's disease [39], diabetes [40, 41], cerebral amyloid angiopathy [42], and ESRD with maintenance HD [35]. Thus, QSM stands as a potential indicator capable of shedding light on vascular structural characteristics in NVC analysis.

Our study showed that CKD patients had disturbed NVC patterns at the level of the whole brain and dominant regions of the HIP, basal ganglia, insula, and ANG, especially in the CKD3b‐5 group, which suggested that non‐dialysis patients with CKD experienced a general mismatch between oxygen demand and blood supply [43], resulting in dysfunction within the NVU. Patients in the CKD3b–5 group had significantly reduced CBF‐ReHo in bilateral HIP, compared to both the CKD1–3a group and HC, and showed a significant positive correlation with MOCA. Furthermore, a significant correlation emerged between susceptibility‐ReHo coupling coefficients and MoCA scores in bilateral HIP of the CKD1–3a group. We speculate that in non‐dialysis patients with CKD, uremia neurotoxin, structural and functional damage of cerebral vessels, endothelial dysfunction, and other reasons lead to iron deposition, cerebral blood flow disorder, decreased neural activity, NVC disorder, and eventually resulted in cognitive impairment, which may be the potential mechanism of cognitive decline in CKD.

The results of our mediation analysis demonstrated that the relationship between phosphorus and MoCA scores in the CKD1‐3a group was mediated by susceptibility‐ALFF alterations in ANG.R, suggesting that CKD‐associated neurovascular biomarkers can be used in conjunction to monitor the progression of cognitive decompensation. ANG, located at the junction of temporal, parietal, and occipital lobes, is an important brain region involved in episodic memory retrieval and recall. More interestingly, through rich connections with other distributed systems, AG can also act as a processing center for a wide range of cognitive functions, including word reading and comprehension, number processing, semantic processing, memory retrieval, attention, and spatial cognition [44]. Previous studies have shown that hyperphosphatemia causes an increase in parathyroid hormone (PTH), and in the general population, elevated PTH is associated with cognitive decline and an increased incidence of dementia [45]. Based on our results, we speculate that this may be due to the fact that iPTH can cross the blood–brain barrier and PTH receptors are highly expressed in the human brain, and that PTH may have a direct effect on the central nervous system [46], leading to disturbances in neurovascular coupling, which can lead to varying degrees of cognitive impairment. These observations suggest that QSM may be as pivotal as CBF in evaluating cerebrovascular status in patients with CKD, and ANG may be a target for the early occurrence of NVC abnormalities.

## 5 Limitation

There are several limitations in this study that merit consideration. First, the relatively small sample size could potentially restrict the scope of our results, potentially limiting the depth of our understanding of NVC dysfunction in non‐dialysis patients with CKD. Second, our study was conducted as a cross‐sectional investigation within the CKD1–3a and CKD3b–5 groups. In the future, we will conduct a longitudinal study, particularly following up with CKD1–3a stage patients. This would enable a more thorough analysis of the changes in NVC due to the progressive nature of CKD, providing a deeper understanding of the temporal aspects of NVC in this population. Third, our study did not conduct the MoCA score for HC, and we will conduct the same clinical laboratory examination and scale assessment for HC in the future. In addition, this time, we chose to use ALFF and ReHo to assess changes in neural activity, and in the future, we will try other metrics such as degree centrality(DC), functional connectivity (FC), etc.

## 6 Conclusions

In conclusion, our study highlights that altered neural activity in non‐dialysis patients with CKD is particularly prominent within the DMN, while changes in CBF and susceptibility values are concentrated in the HIP and basal ganglia regions. These alterations significantly correlate with cognitive decline in this patient population. Crucially, we have identified reduced NVC coefficients, including CBF‐ALFF, CBF‐ReHo, and susceptibility‐ALFF, both at the global and regional levels in non‐dialysis patients with CKD. These findings suggest that neurovascular uncoupling may represent a potential underlying mechanism contributing to cognitive decline in non‐dialysis patients with CKD. Furthermore, the susceptibility‐ALFF coupling coefficient stands out as a promising new candidate biomarker for the early assessment of cognitive decline in this patient group.

## Author Contributions

All authors have contributed to the manuscript. Lijun Song and Hao Wang designed and conducted the study, contributed to the data analysis, and drafted the manuscript. Wenbo Yang, Boyan Xu, Heyu Ding, Han Lv, and Mingan Li collected the data. Pengfei Zhao, Min Li, Zhenghan Yang, Wenhu Liu, Zhenchang Wang, and Xu Liu contributed to the design of the study, provided advice on the data analysis, and revised the manuscript.

## Conflicts of Interest

The authors declare no conflicts of interest.

## Supporting information


Data S1.

## Data Availability

These data will be allowed to be used with the consent of the authors.
