# Supplementary material for: Combination of rs‐fMRI, QSM, and ASL Reveals the Cerebral Neurovascular Coupling Dysfunction Is Associated With Cognitive Decline in Patients With Chronic Kidney Disease
Source: CNS Neurosci Ther. 2024 Dec 5;30(12):e70151. doi: 10.1111/cns.70151 (PMC11621384; doi:10.1111/cns.70151)
Supplement: Supplementary file 1 — Data S1. [file CNS-30-e70151-s001.docx]

**Multimodal MRI data acquisition**

During the scan, all participants were asked to lie flat on their backs, close their eyes, keep their heads still and stay awake while trying to avoid any thinking.The rs-fMRI data was obtained with the following parameters: 200 time points, 28 slices with a slice thickness of 5 mm (1-mm gap), TR = 2,000 ms, TE = 35 ms, FOV = 224mm × 224 mm, FA = 90°, matrix = 64 × 64, and acquisition time = 368 s. High-resolution T1-weighted images was obtained by a 3D brain volume imaging (3D BRAVO) sequence with the following parameters: 192 slices with a slice thickness of 1 mm and no gap, TR = 8.492 ms, TE = 3.276 ms, TI = 450 ms, flip angle (FA) = 15°, FOV = 224mm × 224 mm, and matrix = 256×256. Perfusion imaging was obtained using a 3D-pCASL sequence with a background suppression with the following parameters: 36slices with a slice thickness of 4mm; TR = 4844 ms; TE = 10.5 ms; FOV = 240 × 240 mm; matrix = 128 × 128; labeling duration/post label delay, 2025 ms/2025 ms; number of excitations = 3; and spiral-in readout, 512 sample points with 8 arms. QSM data was obtained using a 3D multi-echo gradient echo sequence using the following parameters: 140 slices with a slice thickness of 1 mm and no gap, TR = 42.3 ms; number of echoes = 8; first TE = 3.5 ms; FA = 12°; FOV = 256mm × 256 mm; and reconstruction matrix = 256 × 256.

**Multimodal data analysis**

***Resting-state functional magnetic resonance image analysis***

The rs-fMRI data were pre-processed using the DPABI V6.0 (http://rfmri.org/dpabi) software package on the MATLAB 2018b (https://www.mathworks.com/) platform with the following steps: (1) convert the DICOM format to NFITI format; (2) remove the first 10 time points; (3) slice timing; (4) head movement correction, reject the subjects with head movement rotation >3°or 3mm, or mean framewise displacement (FD) > 0.5;^1^ (5) spatial normalization, using DARTEL algorithm to make space standardization to Montreal Neurological Institute (MNI) template with the resampling voxel size of 3 mm × 3 mm × 3 mm; (6) regression covariate, head parameters, white matter signal, cerebrospinal-fluid signal, and global signal were carried out as covariates; (7) smooth, a Gaussian smoothing kernel with a full-width half-height value of 6 mm was used to perform spatial smoothing; (8)detrend, remove the linear drift. The average amplitude value of the low-frequency amplitude of the brain (0.01–0.08Hz) was extracted, the ALFF values were analyzed; ReHo maps were generated before smoothing, and the normalized images were filtered (0.01–0.08Hz) and normalized before spatial smoothing. The ReHo maps were obtained as ReHo parameters for statistical analysis.

***CBF calculation and analysis***

The processing of ASL were described in our previous studies^2-4^. Quality control was used to exclude substandard images. ASL difference images were obtained by subtracting the labeled images from the control images with a single-compartment model^5^. The CBF map was estimated by averaging three ASL difference images in combination with a proton density-weighted reference image^6^. SPM12 was used for data preprocessing. The CBF images of the 50 HC were nonlinearly aligned to the positron emission tomography-perfusion template in MNI space by using the nonlinear transformation in statistical parametric mapping 12 (SPM12). The average co-registered CBF map of 50 HC was used as the standard CBF template. CBF maps of all participants were co-registered to the standard CBF template of MNI. Normalization was performed by dividing the CBF of each voxel by the average CBF of the whole brain. Finally, a 6 mm full-width half-maximum Gaussian kernel was used for each normalized CBF image for smoothing (Figure 2).

***QSM image reconstruction and analysis***

The QSM reconstruction method was described in our previous study^3^. The QSM images reconstructed from phase image data were obtained by using the STI Suite version 3.0 software package. First, all QSM images were visually inspected to exclude nonconforming images; Second, the original phase image is unfolded using a Laplace-based phase unfolding method^3^; Third, a spherical mean filtering method was used to increase the radius of the spherical nucleus from 0.6 mm at the brain border to 25 mm at the brain center^7^. Finally, susceptibility maps were obtained from frequency maps of brain tissue using a modified LSQR method^3, 8^. The QSM images were pre-processed using SPM12 and code written in MATLAB 2018b (MathWorks, Natick, MA) with the following flow: First, the T1 structural image of each participant was co-aligned with the first echo amplitude image obtained using a multi-echo gradient echo sequence. Second, the aligned T1 images were normalized to MNI space by using FSL (www.fmri.ox.ac.UK/FL, Version 6.0) and all QSM images were transformed to MNI space. Finally, the normalized QSM images were smoothed using a 6 mm isotropic Gaussian kernel to reduce inter-individual variability^3^ (Figure 2).

***Voxel-wise comparisons of ALFF, ReHo, CBF values and susceptibility values***

Differences in voxel-based ALFF, ReHo, CBF and susceptibility were compared among the three groups after controlling for age, sex education medication and TIV using SPM12.

***Analysis of global-based NVC***

Global coupling of neuronal activity (ALFF, ReHo) and vasculature (CBF and susceptibility values) was analyzed using code written in MATLAB 2018b (MathWorks, Natick, MA). For each participant, four modes of NVC (CBF-ALFF, CBF-ReHO, susceptibility- ALFF value, and susceptibility-ReHo value coefficients) were evaluated at the global level.

***Analysis of region of interest (ROI)-based neurovascular coupling***

Neuronal activity (ALFF, ReHo) and vascularity (CBF and susceptibility values) were analyzed across voxel region coupling using multi-modal image coupling analysis software^9^. The brain was divided into 116 separate regions according to the AAL template.Brain images were multiplied with predefined ROIs to form ROI images. The program package then sequentially calculates the four crosses voxel NVC coefficients (CBF-ALFF, CBF-ReHo, susceptibility-ALFF, and susceptibility-ReHo coefficients) between two ROI images.

**Mediation analysis**

Firstly, a partial correlation analysis with sex, age, education, medication and TIV as covariates was used to analyze the correlation between blood biochemical indicators, imaging indicators (ALFF, ReHo,CBF, susceptibility value), NVC and MoCA and its subsets. According to the partial correlation results, phosphate was the independent variables, MoCA was the dependent variable, and NVC change was the mediator variable. SPSS 25.0 was used for mediation analysis.

**Reference**

**1.** Yan CG, Wang XD, Zuo XN, Zang YF. DPABI: Data Processing & Analysis for (Resting-State) Brain Imaging. *Neuroinformatics.* Jul 2016;14(3):339-351.

**2.** Wang H, Liu X, Song L, et al. Dysfunctional Coupling of Cerebral Blood Flow and Susceptibility Value in the Bilateral Hippocampus is Associated with Cognitive Decline in Nondialysis Patients with CKD. *J Am Soc Nephrol.* Jul 20 2023.

**3.** Wang H, Song L, Li M, Yang Z, Wang ZC. Association between susceptibility value and cerebral blood flow in the bilateral putamen in patients undergoing hemodialysis. *J Cereb Blood Flow Metab.* Mar 2023;43(3):433-445.

**4.** Jin M, Wang L, Wang H, et al. Disturbed neurovascular coupling in hemodialysis patients. *PeerJ.* 2020;8:e8989.

**5.** Hernandez-Garcia L, Lahiri A, Schollenberger J. Recent progress in ASL. *Neuroimage.* Feb 15 2019;187:3-16.

**6.** Xu G, Rowley HA, Wu G, et al. Reliability and precision of pseudo-continuous arterial spin labeling perfusion MRI on 3.0 T and comparison with 15O-water PET in elderly subjects at risk for Alzheimer's disease. *NMR Biomed.* Apr 2010;23(3):286-293.

**7.** Li W, Wu B, Batrachenko A, et al. Differential developmental trajectories of magnetic susceptibility in human brain gray and white matter over the lifespan. *Hum Brain Mapp.* Jun 2014;35(6):2698-2713.

**8.** Li W, Wang N, Yu F, et al. A method for estimating and removing streaking artifacts in quantitative susceptibility mapping. *Neuroimage.* Mar 2015;108:111-122.

**9.** Hu B, Yu Y, Wang W, Cui GB. MICA: A toolkit for multimodal image coupling analysis. *J Neurosci Methods.* Jan 1 2021;347:108962.


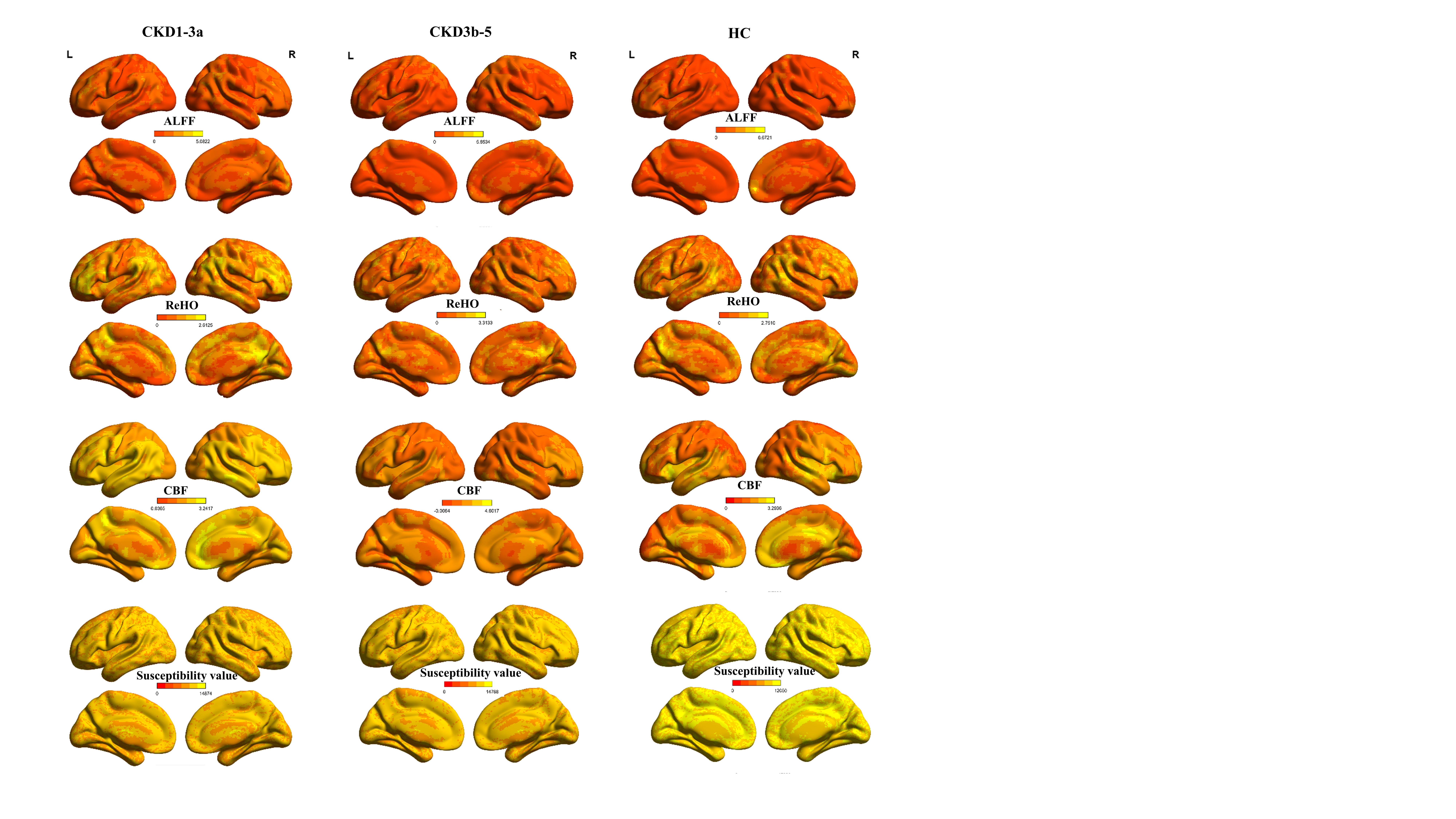


Supplementary Figure 1: Spatial distribution maps of ALFF, ReHo, CBF and susceptibility value in healthy controls and patients with CKD. CKD1–3a, patients with stage 1–3a chronic kidney disease; CKD3b–5, patients with stage 3b–5 chronic kidney disease; HC = healthy control; CBF, cerebral blood flow; ALFF, amplitude of low frequency fluctuations; ReHo, regional homogeneity.


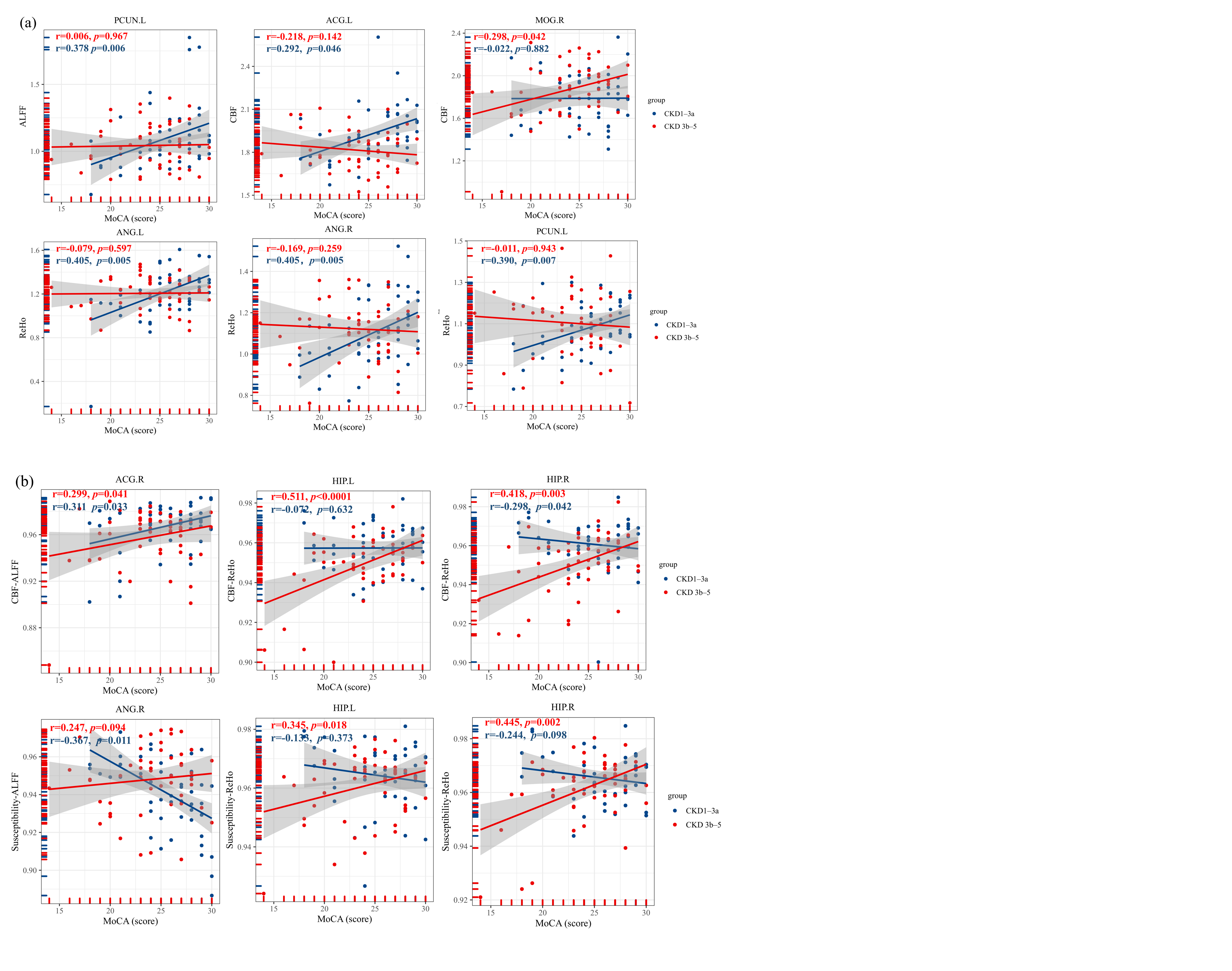


Supplementary Figure. 2. (a) Scatter plot showing the correlation between ROI-based neurovascular indicators and MoCA scores in patients with CKD. (b) Scatter plots of whole-brain and ROI-based correlations of neurovascular coupling metrics with MoCA scores in patients with CKD. Bonferroni corrected was used in multiple comparisons (*P*<0.05/116 = 0.0004) with sex, age, education level, medication and TIV as covariates. TIV, total intracranial volume; CKD, chronic kidney disease; CKD3b–5, patients with stage 5 chronic kidney disease; CKD1–3a, patients with stage 1–3a chronic kidney disease; HC, healthy control; ALFF, amplitude of low frequency fluctuations; ReHo, regional homogeneity; CBF, cerebral blood flow; QSM, quantitative susceptibility mapping; ROI, region of interest; ANG, angular gyrus; ACR, anterior cingulate and paracingulate gyri; HIP, hippocampus; R, right; L, left.
